# Highly Sensitive Acetone Gas Sensor Based on g-C_3_N_4_ Decorated MgFe_2_O_4_ Porous Microspheres Composites

**DOI:** 10.3390/s18072211

**Published:** 2018-07-10

**Authors:** Run Zhang, Yan Wang, Zhanying Zhang, Jianliang Cao

**Affiliations:** 1 The Collaboration Innovation Center of Coal Safety Production of Henan Province, Henan Polytechnic University, Jiaozuo 454000, China; zhangrun0518@126.com; 2 School of Chemistry and Chemical Engineering, Henan Polytechnic University, Jiaozuo 454000, China; 3 State Key Laboratory Cultivation Bases for Gas Geology and Gas Control, Jiaozuo 454000, China; zhangzy@hpu.edu.cn

**Keywords:** g-C_3_N_4_ nanosheet, MgFe_2_O_4_ porous microspheres, composites, acetone, gas sensing

## Abstract

The g-C_3_N_4_ decorated magnesium ferrite (MgFe_2_O_4_) porous microspheres composites were successfully obtained via a one-step solvothermal method. The structure and morphology of the as-prepared MgFe_2_O_4_/g-C_3_N_4_ composites were characterized by the techniques of X-ray diffraction (XRD), field-emission scanning electron microscopy (FESEM), transmission electron microscopy (TEM), thermal gravity and differential scanning calorimeter (TG–DSC) and N_2_-sorption. The gas sensing properties of the samples were measured and compared with a pure MgFe_2_O_4_-based sensor. The maximum response of the sensor based on MgFe_2_O_4_/g-C_3_N_4_ composites with 10 wt % g-C_3_N_4_ content to acetone is improved by about 145 times, while the optimum temperature was lowered by 60 °C. Moreover, the sensing mechanism and the reason for improving gas sensing performance were also discussed.

## 1. Introduction

Acetone, as a highly volatile and flammable organic compound, is widely used in industries or laboratories as a solvent, chemical intermediate and industrial product [1]. Chronic exposure to an acetone atmosphere causes inflammation and may even cause damage to the liver and kidney, while acute poisoning can harm the central nervous system [2]. In addition, acetone is also a widely accepted breath biomarker for type-I and type-II diabetes [3,4]. Thus, fast and timely monitoring of the existence and concentration of acetone is of great importance for human safety and health.

Gas sensors based on metal oxide semiconductors (MOS) are attractive candidates due to their low cost, fast response and easy fabrication [5,6]. Thus, MOS-based gas sensors have been regarded as an important method of monitoring flammable and toxic gases. Up to now, several types of MOSs have been developed as gas-sensing materials to detect acetone, such as In_2_O_3_ [7], WO_3_ [8,9], SnO_2_ [10], La_2_O_3_ [11], Co_3_O_4_ [12], ZnO [13], α-Fe_2_O_3_ [14] and so on. However, the gas-sensing materials have limited properties, some of which are unable to satisfy the needs of practical applications. The development of high performing and stable acetone-sensing materials remains a challenging task.

In recent years, spinel ferrite MFe_2_O_4_ (M = Mg, Co, Ni, Zn, Mn, Cd, etc.) gas-sensing materials have attracted a significant amount of research interest due to their high selectivity and good chemical stability compared to traditional metal oxide semiconductor gas sensing materials [15]. As a magnetic n-type semiconductor, magnesium ferrite (MgFe_2_O_4_) has been used in the research field of gas sensing [16,17,18]. For example, Patil et al. successfully prepared two types of spinel MgFe_2_O_4_ thick films via a sol–gel process, which revealed that the best response to acetone vapor occurred at 350 °C and 450 °C, respectively [19]. However, experimental results have shown that MgFe_2_O_4_ gas sensing materials have obvious shortcomings, such as poor electrical characteristics and high working temperatures.

In order to improve the properties of gas sensors, compounding MOS with high specific surface area 2D nanomaterials has been proven to be an effective way of creating a synergistic effect through these components [20,21]. Li et al. synthesized Zn_2_SnO_4_ nanoparticles/reduced graphene oxide via a solvothermal route and found that the nanocomposites showed good sensitivity to ethanol [22]. Chen et al. synthesized WO_3_ microspheres loaded with small-size Pt-decorated graphene composite, which exhibited a high selectivity and sensitivity to low concentration acetone gas at the operating temperature of 200 °C [9]. As a new two-dimensional (2D) semiconductor, graphitic carbon nitride (g-C_3_N_4_) possesses several advantages, such as high chemical stability, high specific surface area, unique electronic structure, facile preparation and non-toxicity [23,24]. Several previous studies focusing on g-C_3_N_4_ decorated various metal oxide composites proved that g-C_3_N_4_ plays a very important role in the composites. It not only enlarges the specific surface area and prevents agglomeration of metal oxide nanoparticles, but also forms a heterojunction with MOSs and provides new chemical and structural properties [25,26,27]. Cao et al. reported that SnO_2_/g-C_3_N_4_ nanocomposites show a favorable response to ethanol by a facile calcination method [28]. Hu et al. synthesized the 2D C_3_N_4_-tin oxide gas sensors by a one-step method for enhanced acetone vapor detection [29].

In this work, we report the synthesis of g-C_3_N_4_ nanosheet decorated MgFe_2_O_4_ porous microspheres composites via a one-step solvothermal method. With comparison of the gas-sensing properties of MgFe_2_O_4_/g-C_3_N_4_ composites with different g-C_3_N_4_ contents, including the sensitivity, stability and selectivity, we concluded that the properties of MgFe_2_O_4_-based sensors are remarkably improved due to the introduction of g-C_3_N_4_. In particular, the sensor based on 10 wt % g-C_3_N_4_ decorated MgFe_2_O_4_ porous microspheres exhibited the best gas-sensing performance.

## 2. Experimental

### 2.1. Preparation of the MgFe_2_O_4_/g-C_3_N_4_ Composites

All chemicals were of analytical purity and were used without further purification. Graphitic carbon nitride (g-C_3_N_4_) was synthesized by our previous reported method [30,31]. MgFe_2_O_4_/g-C_3_N_4_ composites were prepared by a facile solvothermal process. Typically, a certain mass of g-C_3_N_4_ was dissolved in 80 mL of ethylene glycol with ultrasonic treatment for 2 h. After this, 1.015 g of magnesium chloride hexahydrate (MgCl_2_·6H_2_O, 99.0%), 2.702 g of ferric chloride nonahydrate (FeCl_3_·9H_2_O, 99.0%) and 0.54 g of urea (CO(NH_2_)_2_, 99.0%) were added into the previously dispersed suspension with magnetic stirring for 30 min. Urea is mainly added in order to adjust the pH value of the solution. Finally, the mixture was transferred into a 100-mL stainless-steel Teflon-lined autoclave and kept for 24 h at 200 ℃ in an oven. The product was collected and washed with DI water and ethanol several times, before being finally dried at 60 ℃ for 24 h. The ratio of MgFe_2_O_4_/g-C_3_N_4_ composites was controlled by adjusting the weight of the g-C_3_N_4_ added. According to this process, the MgFe_2_O_4_/g-C_3_N_4_ composite with the 5 wt %, 10 wt % and 15 wt % contents of g-C_3_N_4_ decorated MgFe_2_O_4_ were prepared and marked as MgFe_2_O_4_/g-C_3_N_4_-5, MgFe_2_O_4_/g-C_3_N_4_-10 and MgFe_2_O_4_/g-C_3_N_4_-15, respectively. For comparison, pure MgFe_2_O_4_ without g-C_3_N_4_ nanosheets was prepared by the same method.

### 2.2. Sample Characterization

Powder X-ray diffraction (XRD, Cu-Kα, Bruker-AXSD8) (Bruker, Madison, WI, USA) was used to examine the purity and crystalline of the samples over a 2*θ* range of 10–90°. Field emission scanning electron microscopy (FESEM, Quanta™250 FEG) (FEI, Eindhoven, The Netherlands) and transmission electron microscopy (TEM, JEOL JEM-2100) (JEOL, Tokyo, Japan) were used to analyze the morphologies and structures of the as-prepared samples. Thermal gravity and differential scanning calorimeter (TG–DSC) for the samples of g-C_3_N_4_ and MgFe_2_O_4_/g-C_3_N_4_-10 were recorded on a TA-SDT Q600 (TA Instruments, New Castle, DE, USA) over a temperature range of 30–800 °C at a heating rate of 10 °C min^-1^ under a flowing air atmosphere. The porous features of the samples were characterized by the N_2_ adsorption–desorption measurement (Quantachrome Autosorb-iQ2 sorption analyzer) (Quantachrome, Boynton Beach, FL, USA). Before obtained the measurement, the samples were degassed at 200 °C for more than 12 h. The specific surface area of the sample was estimated by using the Brunauer–Emmett–Teller (BET) method and the pore size distribution was derived from the Density functional theory (DFT) method.

### 2.3. Gas Sensing Property Test

**Figure 1:**
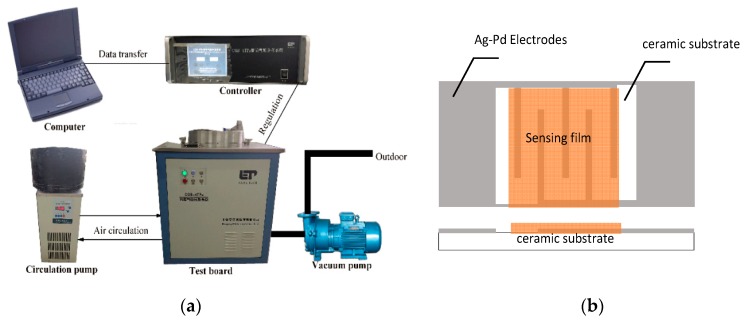
(**a**) The CGS-4TPS gas-sensing test system and (**b**) the gas sensor substrate.

The gas-sensing property tests of the pure MgFe_2_O_4_ porous microspheres and MgFe_2_O_4_/g-C_3_N_4_ composites with different contents of g-C_3_N_4_ were investigated by using an intelligent gas sensing analysis system of CGS-4TPS (Beijing Elite Tech. Co., Ltd. Beijing, China). The fabrication and test process for the sensors is similar to our previously reported method. Figure 1 shows a simple device schematic diagram. The relative humidity is 25% and the temperature is 25 °C in the test chamber during the process of the gas-sensing testing. The response of the gas sensor is defined as follows: Response = R*_a_*/R*_g_* (R*_a_* and R*_g_* were the resistances of the sensor measured in air and in test gas, respectively).

## 3. Results and Discussion

### 3.1. Sample Characterization

**Figure 2:**
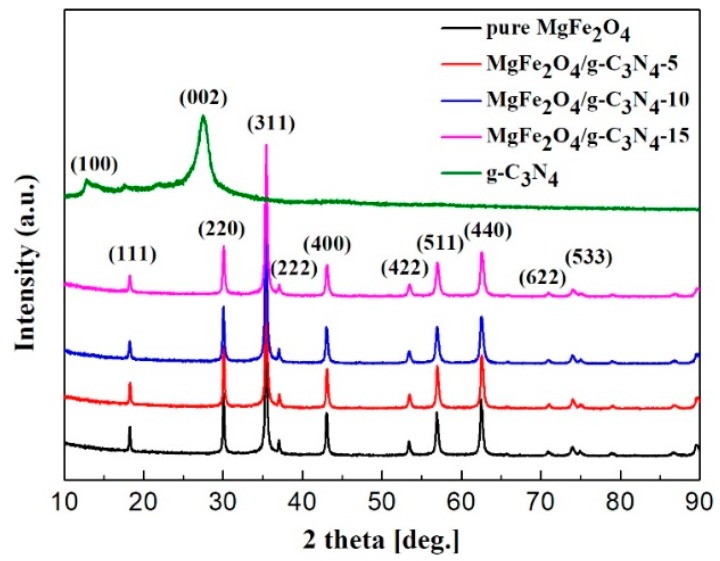
XRD patterns of g-C_3_N_4_, MgFe_2_O_4_ porous microspheres and MgFe_2_O_4_/g-C_3_N_4_ composites with different g-C_3_N_4_ contents.

Figure 2 shows the XRD patterns of g-C_3_N_4_, MgFe_2_O_4_ porous microspheres and MgFe_2_O_4_/g-C_3_N_4_ composites with different g-C_3_N_4_ contents. We found that the diffraction peaks of MgFe_2_O_4_ were consistent with the standard pattern of MgFe_2_O_4_ (JCPDS card No. 17-0464) [32]. For g-C_3_N_4_, a strong peak appears at around 27.71° that corresponds to (002) diffraction plane (JCPDS card No. 87-1526) [33], which is well-known for the melon network. Another peak at 12.81° corresponds to (100) ordering of tri-s-triazine units. However, there is no diffraction peak of g-C_3_N_4_ observed in the curves of MgFe_2_O_4_/g-C_3_N_4_ composites. This may be due to the relatively small content of g-C_3_N_4_ in the composite.

**Figure 3:**
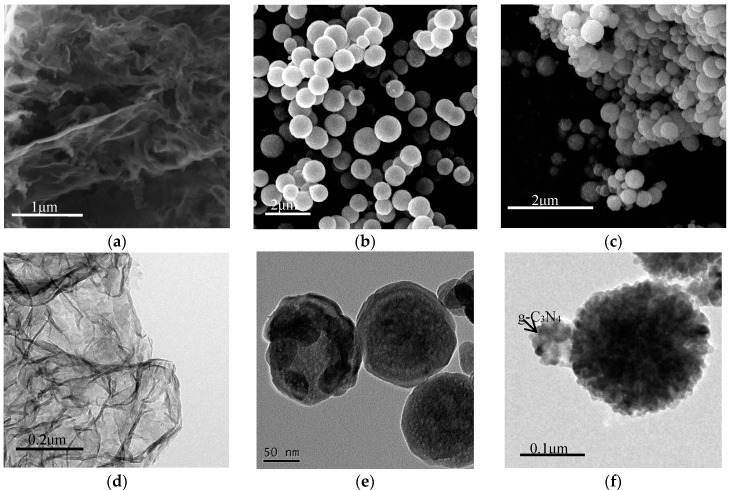
Scanning electron microscope (SEM) images of (**a**) g-C_3_N_4_; (**b**) MgFe_2_O_4_ porous microspheres and (**c**) MgFe_2_O_4_/g-C_3_N_4_ composite; as well as transmission electron microscopy (TEM) images of (**d**) g-C_3_N_4_; (**e**) MgFe_2_O_4_ porous microspheres and (**f**) MgFe_2_O_4_/g-C_3_N_4_ composite.

The morphologies and structures of the as-prepared samples were verified by FESEM and TEM. Figure 3a,d display the SEM and TEM images of pure g-C_3_N_4_. It can be observed that the pure g-C_3_N_4_ possesses a typical lamellar structure with many wrinkles. The SEM and TEM images of the MgFe_2_O_4_ microspheres are shown in Figure 3b,e. From the images, we can see that the prepared MgFe_2_O_4_ consists of very uniform microspheres with a hierarchical structure and a diameter of 200–250 nm. The TEM image shows that hundreds of nanoparticles form the building blocks of the MgFe_2_O_4_ microspheres, leading to the formation of a hierarchical structure and porous features. Figure 3c shows the SEM image of MgFe_2_O_4_/g-C_3_N_4_-10 composite. Compared with Figure 3b, we found that the originally dispersed MgFe_2_O_4_ microspheres were adhered to each other. From Figure 3f, we confirmed the existence of g-C_3_N_4_ and thus, we can conclude that the association phenomenon is due to the introduction of g-C_3_N_4_.

**Figure 4:**
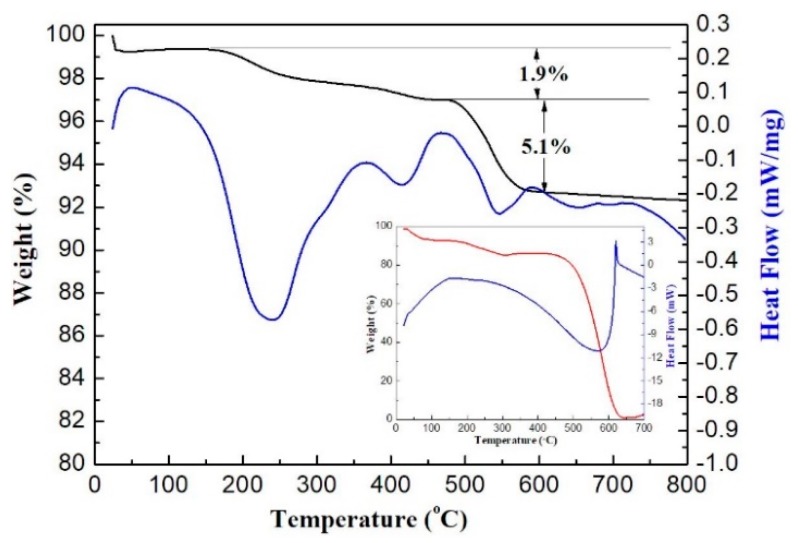
Thermogravimetry–differential scanning calorimeter (TG–DSC) profiles of g-C_3_N_4_ and MgFe_2_O_4_/g-C_3_N_4_-10 composites.

In order to determine the high temperature property of the g-C_3_N_4_ and MgFe_2_O_4_/g-C_3_N_4_-10, TG–DSC analysis was applied. As shown in Figure 4, there are three stages of weight loss in the MgFe_2_O_4_/g-C_3_N_4_ curve according to the peaks of DSC curve. The first stage in the temperature range of 100–300 °C is due to the desorption of adsorbed and trapped water and gas molecules. The reason of the second stage of weight loss between 300 °C and 450 °C is the removal of the primary solvent (ethylene glycol). The total lost weight of these two stages is 1.9%. The third stage corresponds to the temperature range of 475–635 °C in the TG curve of pure g-C_3_N_4_, which is due to the combustion of g-C_3_N_4_ in air (consistent with Figure 4 inset, the TG–DSC analysis of pure g-C_3_N_4_), and the weight loss of this stage is 5.1%. We proved that MgFe_2_O_4_/g-C_3_N_4_-10 composite could work normally without decomposing below the gas sensing test temperature of 475 °C.

**Figure 5:**
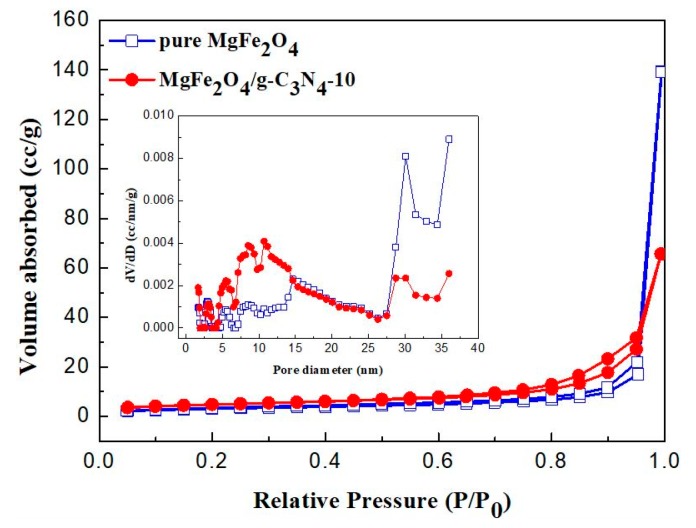
N_2_ adsorption–desorption isotherms and (inset) the corresponding pore size distribution curves of the MgFe_2_O_4_ porous microspheres and MgFe_2_O_4_/g-C_3_N_4_-10 composites.

N_2_-sorption measurements of the as-prepared MgFe_2_O_4_ porous microspheres and MgFe_2_O_4_/g-C_3_N_4_-10 composite were further performed to investigate their specific surface area and porous structure. As shown in Figure 5, the nitrogen adsorption–desorption isotherms of the two samples show a type IV adsorption-isotherm according to the International Union of Pure and Applied Chemistry (IUPAC) classification, which is indicative of a mesoporous structure. The hysteresis loop of MgFe_2_O_4_/g-C_3_N_4_-10 samples belongs to H_3_-type, which demonstrates the presence of a laminated structure with narrow slits formed by MgFe_2_O_4_ microspheres and g-C_3_N_4_ sheet. Figure 5 (inset) depicts the pore size distribution curves of MgFe_2_O_4_ microspheres and MgFe_2_O_4_/g-C_3_N_4_-10 composite. It can be seen from Figure 5 that the pore diameter of MgFe_2_O_4_ and MgFe_2_O_4_/g-C_3_N_4_-10 composite is concentrated in the ranges of about 30–40 nm and 5–15 nm, respectively. This is in agreement with the TEM analysis results. The result illustrates that g-C_3_N_4_ fills the relatively large pores between the MgFe_2_O_4_ microspheres. The BET surface areas of MgFe_2_O_4_ and MgFe_2_O_4_/g-C_3_N_4_-10 samples were calculated to be 11.0 m^2^·g^−1^ and 16.8 m^2^·g^−1^, respectively.

### 3.2. Gas Sensing Property

**Figure 6:**
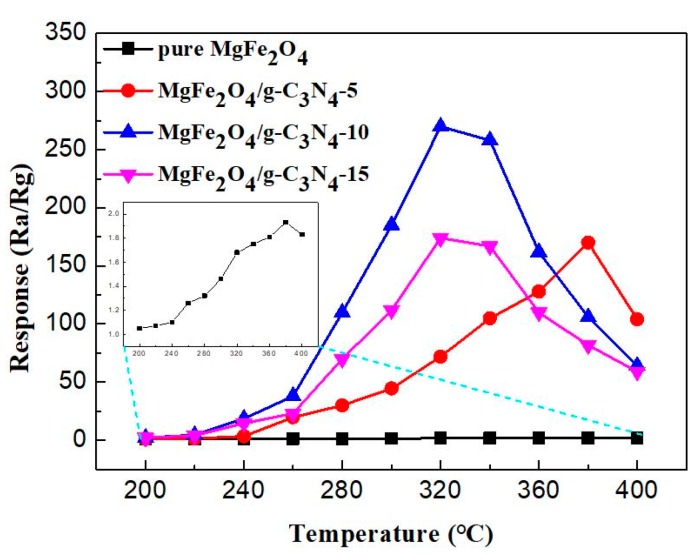
Response values of the sensors based on pure MgFe_2_O_4_, MgFe_2_O_4_/g-C_3_N_4_-5, MgFe_2_O_4_/g-C_3_N_4_-10 and MgFe_2_O_4_/g-C_3_N_4_-15 composites to 500 ppm acetone as a function of operating temperature.

It is well known that the working temperature greatly influences the gas-sensing performance of MOS-based sensor because the gas adsorption and desorption and surface reaction kinetics are all closely related with the working temperature. Figure 6 shows the response of MgFe_2_O_4_ porous microspheres and MgFe_2_O_4_/g-C_3_N_4_ composites-based sensors to 500 ppm acetone at different operating temperatures. It can be seen from Figure 6 that all response curves of sensors exhibit “increase–maximum–decrease” trends with increasing temperature. Comparing different curves, the results demonstrate that the introduction of g-C_3_N_4_ into MgFe_2_O_4_ can greatly enhance the sensor’s response to acetone, with the best g-C_3_N_4_ content in MgFe_2_O_4_/g-C_3_N_4_ composites being 10 wt %. The MgFe_2_O_4_/g-C_3_N_4_-10-based sensor exhibits the highest response value to acetone (which is 275 at 320 °C). Meanwhile, the highest response of the sensor based on pure MgFe_2_O_4_ porous microspheres is 1.9 at 380 °C. Compared with the MgFe_2_O_4_-based sensor, the maximum response of the sensor based on MgFe_2_O_4_/g-C_3_N_4_-10 is improved by about 145 times and the optimum temperature is lowered by 60 °C.

**Figure 7:**
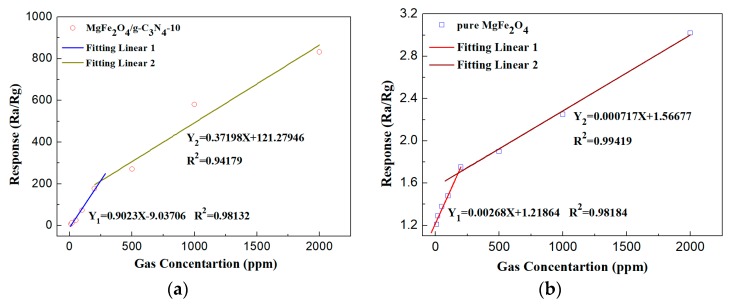
The response value of the sensors based on (**a**) MgFe_2_O_4_/g-C_3_N_4_-10 and (**b**) MgFe_2_O_4_ porous microspheres to the varied concentrations of acetone at the working temperature of 320 °C and their fitting linear.

Figure 7a,b illustrate the response value curves of the pure MgFe_2_O_4_ porous microspheres and MgFe_2_O_4_/g-C_3_N_4_-10-based sensors to varied concentrations of acetone under the working temperature of 320 °C. With an increase in acetone concentration, more acetone molecules can adsorb to the materials’ surface, inducing a rise in the response value. According to the difference of acetone concentration range, two linear relationships were fitted respectively. The fitting linear relationships between the response value and acetone concentration are shown in Figure 7a,b, which provides a possibility for accurately monitoring acetone concentration. The responses of the sensor based on MgFe_2_O_4_/g-C_3_N_4_-10 towards 500, 1000 and 2000 ppm acetone were 271.1, 580 and 832, respectively. Meanwhile, the response values of MgFe_2_O_4_ porous microspheres based sensor were 1.8, 3.2 and 3.0, respectively. The results proved that the degree of gas sensor promotion is more obvious with an increase in acetone concentration. According to the IUPAC definition, the limit of detection (LoD) = 3(Noise_rms_/slope) [34]. The sensor noise was extracted from the root-mean-square (rms) deviation of the response fluctuation using 30 points at the baseline without target gas and the slope was calculated from the linear part in Figure 7. The limit of detection of sensor is determined to be 30 ppb.

**Figure 8:**
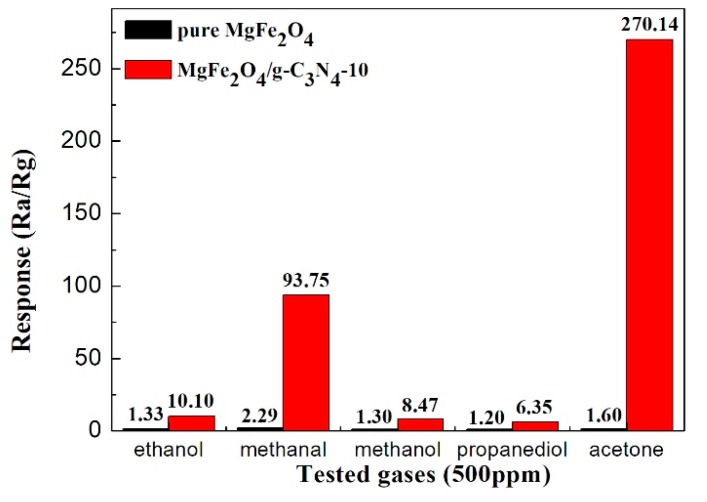
Response values of the sensors based on MgFe_2_O_4_ and MgFe_2_O_4_/g-C_3_N_4_-10 to 500 ppm of different types of tested gas at working temperature of 320 °C.

Figure 8 depicts the response values of the MgFe_2_O_4_/g-C_3_N_4_-10-based sensor and the MgFe_2_O_4_ porous microspheres-based sensor to different gases with the same concentration (500 ppm) at 320 °C, including ethanol, methanal, methanol, propanediol and acetone. Obviously, it reveals that the sensor based on MgFe_2_O_4_/g-C_3_N_4_-10 has admirable selectivity to acetone than to other gases compared to the MgFe_2_O_4_ porous microspheres-based sensor at 320 °C. This selective test results indicates that the MgFe_2_O_4_/g-C_3_N_4_ composite could be a good candidate for the selective detection of acetone.

**Figure 9:**
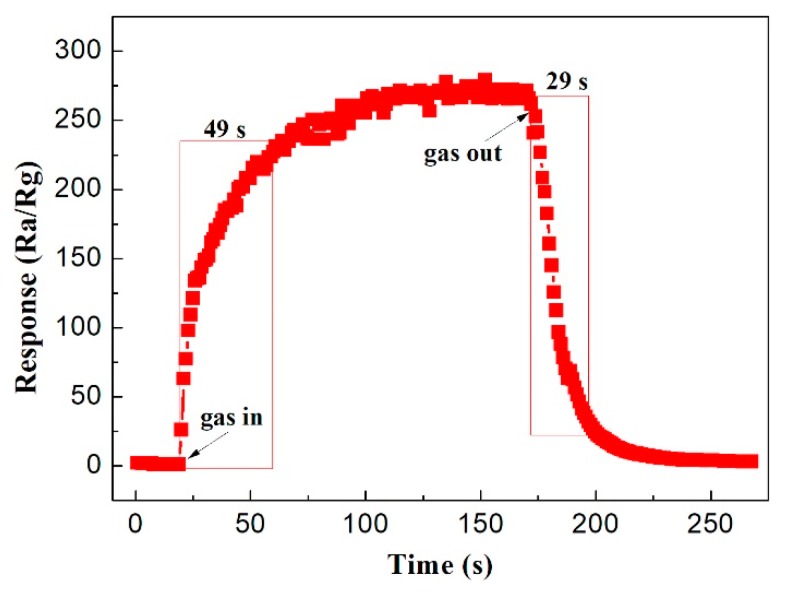
Response–recovery time curve of MgFe_2_O_4_/g-C_3_N_4_-10-based sensor to 500 ppm acetone at working temperature of 320 °C.

Response and recovery times are the important parameters of gas sensors, which are defined as the time required to reach 90% of the change of sensor resistance. The curve of MgFe_2_O_4_/g-C_3_N_4_-10-based sensor to 500 ppm acetone at a working temperature of 320 °C is shown in Figure 9. The response time and recovery time were calculated to be 49 s and 29 s, respectively. The result indicates that the gas sensing material of MgFe_2_O_4_/g-C_3_N_4_-10 possesses good response–recovery characteristics to acetone and could meet the requirements of practical applications.

**Figure 10:**
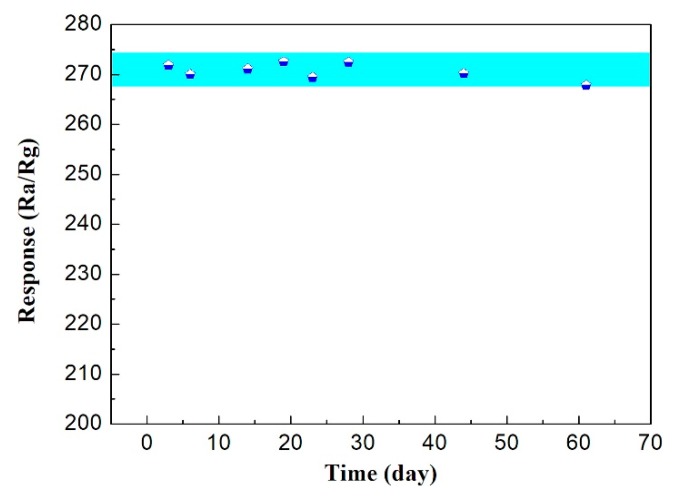
Stability measurement of the sensor based on MgFe_2_O_4_/g-C_3_N_4_-10-10 to 500 ppm acetone at 320 °C.

In order to evaluate the repeatability and stability of the gas sensing properties of MgFe_2_O_4_/g-C_3_N_4_-10 material over time, the sensor responses to 500 ppm of acetone were measured during a period of 60 days and the sensor structure was prepared by using the same drop coating method. Figure 10 displays the long-term stability test results of MgFe_2_O_4_/g-C_3_N_4_-10-based sensor in 60 days. There is a small change within a certain range in the response of sensor from this graph, which further confirms that the MgFe_2_O_4_/g-C_3_N_4_-10-based sensor might have a practical application based on its good long-term stability.

### 3.3. Gas Sensing Mechanism

A modulation model based on the electronic depletion layer [35,36] can explain the gas sensing property. It is well known that MgFe_2_O_4_ and g-C_3_N_4_ are n-type semiconductors. The gas sensitivity of the sensor based on n-type semiconductor material essentially originates from the change of its electric resistance when the sensor is exposed to different gas atmospheres. Taking MgFe_2_O_4_ as an example, when the sensor is exposed to air, O_2_ will be adsorbed on the surface of MgFe_2_O_4_. The oxygen molecules act as electron acceptors from the conduction band and form surface absorbed oxygen anions, such as O_2_^−^, O^−^, and O^2−^ (Equations (1)–(3)). This leads to the formation of a relatively thick electronic depletion layer, which results in an increase in the width of the potential barrier and results in the high resistance of the sensor (R*_a_*). When the sensors are exposed to acetone, the former oxygen anions absorbed on the surface of material will react with the reducing gas (Equations (4)–(6)) and the trapped electrons by absorbed oxygen anions are released back to the conduction band. The resistance of sensors (R*_g_*) can be thus decreased, which accompanies the decrease in the width of the potential barrier.

(1) $$O_{2}{}_{\left(\mathrm{ads}\right)}+e^{-}\to O_{2}^{-}{}_{\left(\mathrm{ads}\right)}$$

(2) $$O_{2}^{-}{}_{\left(\mathrm{ads}\right)}+e^{-}\to 2O_{\left(\mathrm{ads}\right)}^{-}$$

(3) $$O_{\left(\mathrm{ads}\right)}^{-}+e^{-}\to O_{\left(\mathrm{ads}\right)}^{2-}$$

(4) $$CH_{3}COCH_{3}{}_{\left(\mathrm{ads}\right)}+4O_{2}^{-}{}_{\left(\mathrm{ads}\right)}\to 3CO_{2}+3H_{2}O+4e^{-}$$

(5) $$CH_{3}COCH_{3}{}_{\left(\mathrm{ads}\right)}+8O_{{}_{\left(\mathrm{ads}\right)}}^{-}\to 3CO_{2}+3H_{2}O+8e^{-}$$

(6) $$CH_{3}COCH_{3}{}_{\left(\mathrm{ads}\right)}+8O_{\left(\mathrm{ads}\right)}^{2-}\to 3CO_{2}+3H_{2}O+16e^{-}$$

In this work, we found that MgFe_2_O_4_/g-C_3_N_4_-baesd sensors exhibit better acetone sensing properties than pure MgFe_2_O_4_-based sensors. One of the possible reasons for the improved gas performance may be attributed to the heterojunction between the MgFe_2_O_4_ microspheres and g-C_3_N_4_ nanosheet. When the composite sensor was exposed to air, the process of electrons inflow from a component to another. The existence of heterojunction could lead to a higher potential barrier, which further results in an obvious increase in the sensor resistance (R*_a_*). In contrast, due to the surface redox reaction between oxygen anions and acetone molecules, the potential barrier is decreased and the sensor resistance (R*_g_*) is decreased correspondingly. Meanwhile, the larger surface contributes to more oxygen molecules and acetone molecules being adsorbed on the surface of sensor, which could further enhance the redox reaction between acetone molecules and absorbed oxygen anions.

## 4. Conclusions

In summary, we reported a high sensitivity acetone sensor based on MgFe_2_O_4_/g-C_3_N_4_ composite, which was synthesized via a one-step solvothermal method. By analyzing the given results of XRD, FESEM, TEM, TG–DSC and N_2_-sorption, we proved the existence of g-C_3_N_4_ nanosheet in the MgFe_2_O_4_/g-C_3_N_4_ composite. Due to the introduction of g-C_3_N_4,_ the gas sensing property of MgFe_2_O_4_-based sensor is remarkably improved. Among these composites with different g-C_3_N_4_ contents, the MgFe_2_O_4_/g-C_3_N_4_-10-based sensor exhibited the eximious sensing performance to acetone, such as high sensitivity and selectivity, quick response and recovery as well as favorable stability. The as-prepared MgFe_2_O_4_/g-C_3_N_4_ composite could be a promising candidate for practical applications requiring highly sensitive acetone gas sensors.
